# Hemodialysis Timing Influences Sleep Profile

**DOI:** 10.34067/KID.0000000768

**Published:** 2025-05-29

**Authors:** Mario Bonomini, Gianpaolo Reboldi

**Affiliations:** 1 Department of Medicine and Aging Sciences, “G. d’Annunzio” University, Chieti, Italy; 2 Department of Medicine and Surgery, University of Perugia, Perugia, Italy

**Keywords:** ESKD, hemodialysis, quality of life

Sleep disturbances are a common and major concern in patients undergoing chronic hemodialysis. Sleep alterations in the hemodialyzed population include quality, duration, structure, circadian rhythms, and specific sleep disorders insomnia being the most frequent.^1^ A recent systematic review and meta-analysis including 93 articles reported a pooled prevalence of poor sleep quality and insomnia of 68% and 46%, respectively, in adult hemodialysis patients.^2^ These rates were much higher than the prevalences in the control populations with normal renal function and were significantly reduced in kidney transplantation.^2^ Sleep disorders in patients on hemodialysis are associated with chronic fatigue and poor overall quality of life and may increase the risk of cardiovascular events and death.^3^ Notably, patients receiving hemodialysis have ranked sleep difficulty as one of the major threats to their quality of life.^4^

**Figure 1:**
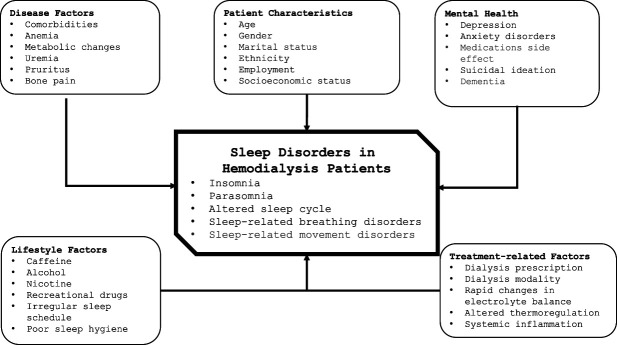
Potential contributing factors to sleep disorders in patients on maintenance hemodialysis.

The pathogenic mechanism(s) of sleep disturbances in hemodialysis patients remain unclear, and it is most likely multifactorial.^1^ Alleged etiologic causes are shown in Figure 1.

In patients receiving in-center hemodialysis, early morning or late evening shifts might also challenge the maintenance of a regular sleep/wake routine.^5^ In this issue of *Kidney360*, Han *et al.*^6^ investigated both natural and hemodialysis-imposed temporal patterns of nocturnal sleep in patients on maintenance hemodialysis. This study has some unique characteristics, such as the large patient population and the sleep monitoring for up to 1 year. Indeed, previous studies on the same topic were generally conducted in a small number of patients and with a short follow-up time.

Sleep monitoring can be performed by using several different tools. Polysomnography is reported as the gold standard to diagnose sleep disorders, although it may be burdensome for the patient and expensive requiring a special sleep facility. Subjective self-report survey and sleep diary are simple and economical methods, but of limited value since often incomplete and inaccurate while not assessing architecture and stages of sleep. A wrist actigraphy device can be used to evaluate sleep patterns at home, but they tend to overestimate the duration of sleep and lack the assessment capability of sleep stage.

In Han's study,^6^ sleep monitoring was performed using commercially available wearable activity trackers (Fitbit Charge 2). The digital health revolution is fueling the development of wearable technology, which is resulting in accurate, sophisticated, and relatively inexpensive devices that can collect data on sleep quality and quantity. Wrist-worn devices seem like an optimal choice to longitudinally study sleep features in free-living conditions over consecutive nights. Han *et al.*^6^ used a recent generation device model, developed through modifications and refinements to early-generation models, which enables to estimate, in addition to sleep parameters and stage, also wake and sleep time.

The study cohort^6^ initially included 109 patients (age 54±12 years, 73% men, 23% diabetic) on thrice weekly hemodialysis, and 72 patients (66%) completed the year-long observation period, yielding an analytical cohort of 14,190 patient-days. Notably, only patients who had a smartphone or a device (computer, tablet, *etc.*) which could support the use of the wearable activity tracker were enrolled. The authors found that nocturnal sleep duration was much shorter than recommended in most patients on hemodialysis, although with large intraindividual variation (from <2 to over 9 hours). The timing of the dialysis procedure had a detrimental effect on nocturnal sleep duration, especially for early starters, while patients slept some 28 minutes more on Sundays compared with other days, irrespective of dialysis schedule. In univariate analyses, male sex, elevated BP, higher creatinine, and lower equilibrated Kt/V were significantly associated with shorter sleep duration. However, only BP and creatinine remained a significant predictor of nocturnal sleep duration in multivariable analysis. A limitation of the study results stems from the multivariable model being restricted to demographics and a few clinical predictors, preventing the investigation of other influential factors with a known effect on sleep duration (Figure 1). Nonetheless, the study offers a valuable and robust insight into nocturnal sleep disturbances in hemodialysis patients and may provide useful information for a personalized sleep medicine approach, leveraging on patient characteristics and advanced technologies for improved diagnosis, treatment, and management.

Although there is an extensive and growing number of reviews on nonpharmacologic and pharmacologic approaches to treat sleep disorders and improve sleep quality in the uremic population, the small number of primary studies and the high overlap between reviews, hampers to draw definitive conclusions on the effectiveness of interventions.^7^ Cognitive-behavioral therapy for insomnia (CBT-I) is recommended by clinical practice guidelines as the first-line intervention for insomnia, but is still largely inaccessible to hemodialysis patients, although CBT-I delivered by telehealth might help to overcome barriers to access. Other nonpharmacologic interventions, including aromatherapy, yoga, music, muscle relaxation, and nurse-led management, showed promising results, but the evidence is limited to a single meta-analysis.^7^ There are limited data on the efficacy and safety of commonly used sleep medications in patients undergoing hemodialysis, in terms of drug selection, dosage, and length.^5^ A recent randomized, double-blinded trial in patients undergoing hemodialysis with mild or moderate chronic insomnia examined the efficacy of trazodone, an attractive option in these patients since it is metabolized in the liver and does not require dose adjustments in kidney failure.^8^ The changes in the Insomnia Severity Index score after 6 weeks with trazodone or CBT-I proved not significantly different compared with placebo. Moreover, the incidence of serious cardiovascular events was higher in the trazodone-treated group.^8^ The study underscores the absence of evidence-based modalities to treat insomnia in hemodialysis population and the need for effective novel interventions.

Han's article^6^ shows that timing of the hemodialysis procedure can adversely influence the temporal patterns of nocturnal sleep duration, which may have clinical implications. Patients beginning the hemodialysis session before 8 AM (early starters) slept significantly less than patients starting hemodialysis later. This effect was quite relevant in the nights before the scheduled hemodialysis procedure, early starters sleeping on average 86 minutes less than the late starters. The dialysis treatment imposed a greater degree of sleep/wake disturbance in early starters. Thus, hemodialysis patients with proven sleep disturbances could be dialyzed later in the day to improve their sleep duration and quality. Although this may not be easily feasible because of current health care services and overworked dialysis facilities, it would represent a tailored therapeutic approach in specific patient population of potential benefit.

Sleep quality is of paramount importance to daytime physical and cognitive performance, and sleep deficiency is now recognized as an independent and significant cardiovascular risk factor alongside traditional risk factor.^9^ Almost half of the patients undergoing maintenance hemodialysis experience sleep difficulty, but most of them do not receive any treatment,^5^ in part because of a paucity of evidence. Screening sleep symptoms is suggested as a first step to support the management of symptom burdens in patients undergoing hemodialysis, and a multidisciplinary approach seems essential to identify the optimal means for diagnosing and managing sleep complications in these patients.^10^ There is the need for robust, high-quality research to better define the efficacy and applicability of interventions in improving sleep health in the dialysis population.^7^ Future studies should also focus on the determinants of sleep from the patients' perspectives, an area which remains largely unexplored,^7^ yet of crucial importance for developing appropriate targeted measures to sleep disorders in patients undergoing hemodialysis.
